# Hydrogen Bond Acceptors Dramatically Promote Fructose Dehydration and Stability of Furanics Like 5-Hydroxymethylfurfural

**DOI:** 10.3390/ijms27177836

**Published:** 2026-09-01

**Authors:** Benjamín Torres-Olea, Gabriela Rodríguez-Carballo, Isamar González-Díaz, Cristina García-Sancho, Ramon Moreno-Tost, Pedro Jesús Maireles-Torres

**Affiliations:** 1 Departamento de Química Inorgánica, Cristalografía y Mineralogía, Facultad de Ciencias, Universidad de Málaga, Campus Universitario de Teatinos s/n, 29071 Málaga, Spainrmtost@uma.es (R.M.-T.);; 2 Instituto de Materiales y Nanotecnología (IMANA), Facultad de Ciencias, Universidad de Málaga, Campus Universitario de Teatinos s/n, 29071 Málaga, Spain; 3 Catalysis and Energy Materials Group, IMDEA Materials Institute, Calle Eric Kandel 2, Getafe, 28906 Madrid, Spain; 4 Instituto Interuniversitario de Investigación en Biorrefinerías (I3B), Universidad de Málaga, Edificio de Investigación Ada Byron, Arquitecto Francisco Peñalosa, 18, Campanillas, 29010 Málaga, Spain

**Keywords:** 5-hydroxymethylfurfural, 5-ethoxymethylfurfural, deep eutectic solvents, catalyst, heterogeneous catalysis, fructose

## Abstract

In this work, it has been demonstrated that the use of hydrogen bond acceptors (HBAs) within the context of deep eutectic solvents (DESs) can be beneficial for fructose dehydration in ethanol to obtain high yields of 5-hydroxymethylfurfural (HMF) and 5-ethoxymethylfurfural (EMF). In this research, several commercial catalysts were screened in the dehydration of fructose under reflux in ethanol. Sulphonated polymers exhibited excellent performance, while zeolites only achieved low yields of HMF due to low acidic strength, rendering them unable to perform the dehydration and etherification of fructose and its subsequent products in the assayed conditions. The addition of an HBA in the form of tetraethyl ammonium bromide (TEAB) was essential to facilitate the dehydration of fructose with zeolites (up to 19% HMF yield with TEAB). In addition, the use of TEAB improved the observed yield of HMF twofold during the reaction when the sulphonated polymer Purolite PD206 was employed (34% with TEAB and 15% HMF yield without TEAB). The investigation of different HBAs showed that tetraethylammonium bromide (TMAC) produced the most promising results, achieving a furanic compound yield of up to 75% after 3 h of reaction at 120 °C, enhancing fructose dehydration kinetics and favoring the stability of furanic rings in the reaction medium.

## 1. Introduction

Current concerns about the supply of liquid fuels and depletion of fossil sources have prompted the search for renewable, high-energy-density chemicals to replace fossil fuels. In this sense, biomass waste is a renewable carbon source produced in huge quantities, on the order of billions of tons yearly, and it possesses different heavily functionalized fractions, mainly cellulose, hemicellulose, and lignin [1]. The introduction of renewable carbon sources into the chemical industry is an unavoidable step in the long term, with eventual decoupling of the economy from fossil resources.

For this reason, different biomass fractions have been investigated for the production of highly versatile chemicals. For example, C6 saccharides can be dehydrated into the building block 5-hydroxymethylfurfural (HMF). This compound has emerged as an important platform chemical within biorefineries, and it is a versatile precursor that can be used for the production of new polymeric materials and provide bio-based routes for the production of widely employed monomers, pharmaceuticals, surfactants, and biofuels [2,3,4]. Despite its potential, the efficient transformation of biomass into HMF and valuable chemicals remains a challenge in green chemistry. The large number of functional groups in saccharides and the instability of the HMF facilitate multiple secondary reaction processes that frequently decrease selectivity. This issue, combined with a lack of engineering research on scaling and bio-based chemical integration, has hindered the transition from laboratory-scale valorization procedures into industrial-scale production of HMF [2].

Fructose is a ketose that has frequently been employed as a starting point in the production of HMF. Fructose dehydrates easily with the mediation of Brønsted acidity, leading to higher HMF yields than when aldoses (glucose and galactose) are used as substrates [5,6]. Due to favorable intermediate stabilization effects, dimethylsulphoxide (DMSO) has been thoroughly exploited for fructose dehydration, frequently leading to very high selectivity and yields. Using fructose as a substrate, Yang et al. utilized Amberlyst-15 in a mixture of DMSO and methanol to produce 5-methoxymethylfurfural directly from fructose at 100 °C for 10 h. As a result, 80% 5-methoxymethyl furfural was reported in a 2:1 methanol:DMSO solvent [7]. Wang et al. employed an MOF synthesised using a Ce-based MOF in an ethanol:DMSO system. A final yield of 78.4% of furanic compounds could be achieved after 1 h at 160 °C [6]. However, efforts are being made in the dehydration of fructose and the production of valuable biofuels in the absence of DMSO, which is a toxic organic solvent for which its separation from HMF is difficult due to the high boiling point of DMSO.

Ionic liquids (ILs) have shown great potential for the valorization of saccharides, but their toxicity, limited stability, and cost make it impossible to scale their use to the industrial level [8]. Deep eutectic solvents (DESs) have emerged as versatile, multifunctional media, gaining traction in catalytic settings, such as the dehydration of saccharides into HMF [9]. DESs have permitted the obtention of high HMF yields under milder and more sustainable reaction conditions than those allowed by mineral acids. Very importantly, DESs change the dielectric constant of the medium, and they can act as catalysts by lowering the activation energy of intermediates [10]. DES for the production of HMF and EMF usually include a hydrogen bond acceptor (HBA), which is typically a quaternary ammonium salt, such as tetraethyl ammonium bromide (TEAB) or choline chloride (ChCl), and a hydrogen bond donor (HBD), usually organic acids or polyols [5,8]. An example of this was the work of Mankar et al., who combined the use of ChCl with lactic acid to generate a DES to produce HMF from fructose in a water:acetone mixture [11]. Their results suggested that microwave radiation was key in the selective activation of fructose and its subsequent dehydration, achieving an HMF yield of 87.2% after 30 min at 140 °C. Zuo et al. investigated the activity of several DESs produced from a combination of ChCl with different HBD molecules [12]. The combination of ChCl with betaine hydrochloride and the introduction of Al_2_O_3_ as a cocatalyst, adding Lewis acidity to the reaction environment, promoted the dehydration of starch into HMF with a maximum yield of 67.1% after 2 h at 140 °C. Other authors have also explored the use of saccharides both as a substrate and as a component of DES for fructose dehydration. For example, Zhang et al. used a tricomponent DES, where the used HBA was choline chloride and the HBD was a mixture of the substrate utilized (e.g., fructose, glucose) and boric acid, which also acted as the catalyst [13]. After 1 h at 120 °C, an HMF yield of 44% was obtained. Thanheuser et al. prepared a self-consuming DES composed of ChCl-Fructose 5:1 [14]. This system used acetonitrile as the extracting phase and H_4_SiW_12_O_40_ as the catalyst. After 12 min at 80 °C, an HMF yield of 70% was achieved. Therefore, the polar environment that is generated when fructose acts as an HBD in a self-consuming DES offers an environment that leads to the selective transformation of the saccharide into competitive HMF yields.

Moreover, HMF can be etherified in ethanol medium to generate 5-ethoxymethylfurfural (EMF). This compound is highly miscible with current fuels, and both its stability and energy density are similar to diesel and gasoline (8.7 kWhL^−1^) [5,15]. Moreover, it has a very high boiling point (235 °C), a high cetane number, stability with respect to oxidation, and reduces the emission of NO_x_ and SO_2_ particles [5]. The production of EMF faces similar challenges to that of HMF, and several strategies have been followed to improve yields and selectivity while keeping economic and environmental costs low. For example, different cosolvents have been employed together with ethanol; the use of biphasic systems, ionic liquids, and DESs has also been evaluated. For example, Naz et al. investigated several acidic ionic liquids in the transformation of wheat husk into EMF [16]. Using a 1,4-diazobicyclo[2.2.2]octane (DABCO)-based ionic liquid, Naz et al. attained a 66% yield of EMF and 34% HMF after 7 h at 130 °C from fructose, while using a pyridinium-based IL permitted the obtention of a 40% EMF yield directly from wheat husk after 130 °C in only 6 h. Gawade et al. transformed fructose at 70 °C after 3 h of fructose treatment, assisted by microwave radiation using a ChCl-oxalic acid DES, yielding an EMF yield of 74% [17]. On the other hand, Quereshi et al. produced EMF using tungsten disulphide as a catalyst assisted by microwaves, resulting in a 62% EMF yield from fructose after 15 min at 160 °C [4].

In this article, we report the efficient transformation of fructose into HMF and EMF using ethanol in a novel, self-consuming deep eutectic solvent (DES) system. The key novelty of this work lies in the dual role of fructose, which acts simultaneously as a reactant and a structural component of the DES when paired with TEAB as the hydrogen bond acceptor (HBA). Through the evaluation of diverse catalysts, including sulfonated polymers and protonic zeolites, we demonstrate that the presence of the HBA dramatically enhances both the dehydration rate and the selectivity toward HMF, which is then progressively etherified into EMF. Furthermore, by systematically investigating the influence of the HBA’s cation and anion, this study provides critical mechanistic insights into how these components dictate reaction pathways. Our results, particularly using the sulfonated polymer Purolite PD206, underscore the superior efficacy of self-consuming DES to promote complex biomass valorization under remarkably mild conditions, offering a sustainable and robust platform for the production of high-value chemicals.

## 2. Result and Discussion

### 2.1. Catalyst Screening

Several catalysts were screened in the transformation of fructose into HMF and EMF at 100 °C in ethanol. Specifically, different zeolites in their protonic form, as well as sulphonic acid functionalized polymers, have been investigated, as these catalytic materials possess strong acidity that can promote both the dehydration of fructose and the etherification of HMF into EMF. For their screening, 0.125 g of catalyst was used together with TEAB (fructose:TEAB at a 6:1 molar ratio) to promote the catalytic activity of the investigated materials. A control experiment was also conducted, which included ethanol, fructose, and TEAB, but without the addition of a catalyst (Figure S1). Under these conditions, 16% of fructose was converted in the blank experiment, but both HMF and EMF were not detected. This demonstrates that the acid catalyst is essential to promote the dehydration of fructose into furanic products. The lack of detectable products during the blank experiment suggests that the hexose could have undergone secondary polymerization reactions into undetectable soluble humins.

**Figure 1:**
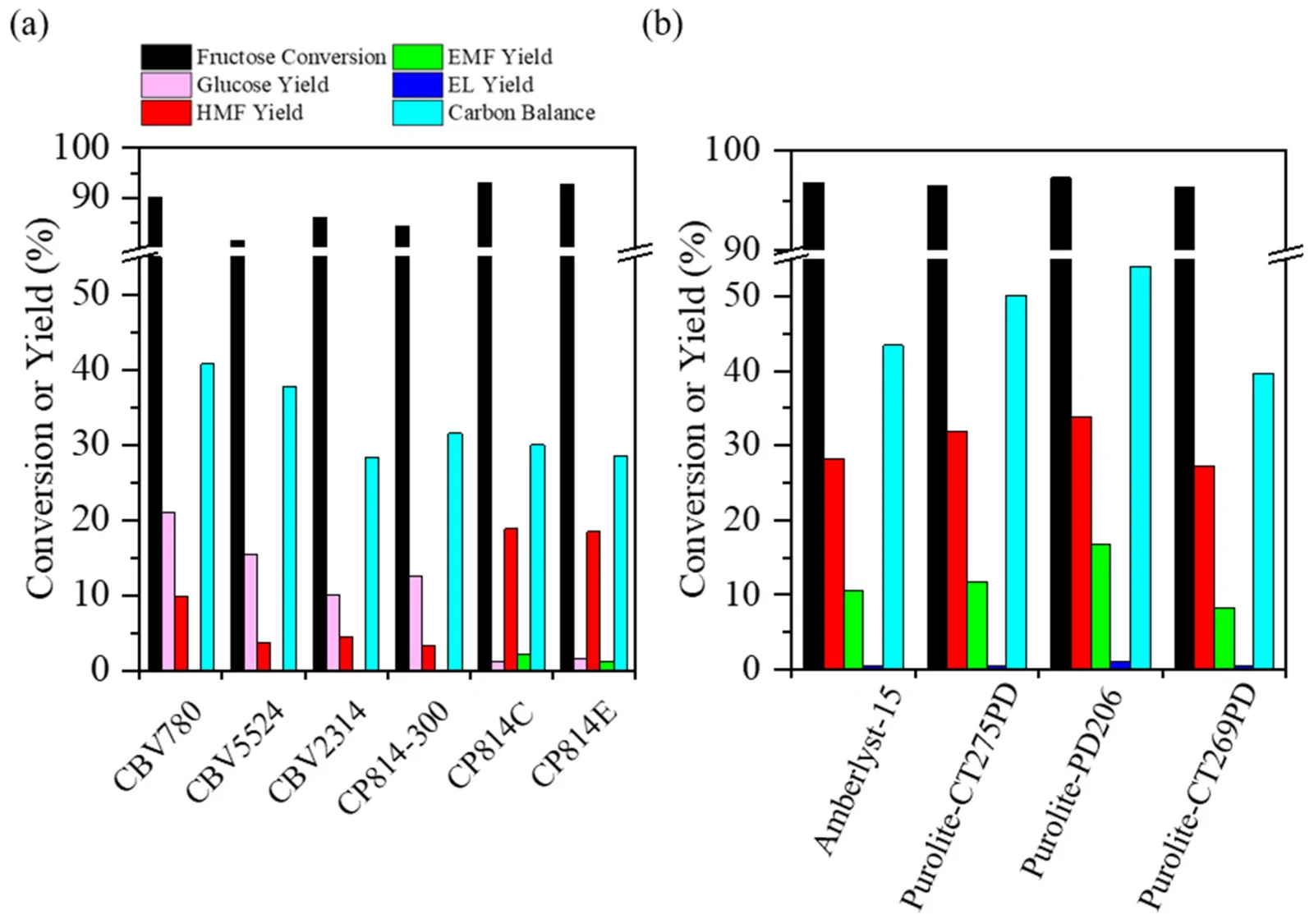
Screening of (**a**) zeolites and (**b**) sulphonic polymers in the process of converting fructose to furans (10 mL of ethanol; 1.25 g fructose; fructose:TEAB at a molar ratio of 6:1; 0.125 g catalyst; 100 °C; 5 h).

The zeolites demonstrated very high activity in the transformation of fructose (Figure 1a). All zeolites achieved fructose conversions higher than 80%. Nevertheless, they displayed limited effectiveness in the production of HMF, with yields only above 10% when zeolites CP814C and CP814E were used. Most of the converted fructose underwent secondary reactions, leading to the formation of humins and a consequently poor carbon balance. The main product of the tested zeolites was glucose. Therefore, zeolites preferentially promote the internal hydride transfer, generating the aldose form, which is much more resistant to dehydration than fructose. Very interestingly, we can see that USY and ZSM5 zeolites (UCBV780, CBV5524, and CBV2314) tend to provide higher carbon balances than Beta zeolites with high aluminum content, specifically CP814E and CP814C (Table S1). This is of interest because the results demonstrate that confinement effects inherent to Beta zeolite facilitate fructose transformation into HMF. In fact, CP814C and CP814E were the only materials capable of the etherification of HMF under conditions of refluxing ethanol. Beta zeolites are highly porous materials for which their internal cavities show similar dimensions to hydrated HMF moieties (Table S1), suggesting the stabilization of key intermediates that promote fructose dehydration. On the other hand, while CP814-300 is another Beta zeolite, it has a very low Si:Al ratio of 150 (Table S1) and displays similar activities compared to ZSM5 and USY zeolites. The finding that CP814-300 shows low activity in the transformation from fructose to HMF reveals that the structure itself is incapable of stabilizing intermediates and provides low-energy transition states by itself, but in fact, protonic Beta zeolites require available protons in their structure to promote the transformation. On the other hand, materials that display lower cavity sizes (ZSM5) or larger cavities (USY) promote the isomerization of fructose into glucose, albeit with yields of up to 20% after 5 h under ethanol reflux.

In contrast with the behavior of zeolitic materials, sulfonic polymers achieved the production of HMF and EMF from fructose in all cases (Figure 1b). The four tested sulfonic materials attained fructose conversions higher than 95% in 5 h. The sulfonated polymer Purolite PD206 attained a 34% HMF yield and a 17% EMF yield, a combined yield of 51% of furanics. Furthermore, a 2.35% yield of ethyl levulinate was observed. Purolite-CT275DR was the second best-performing polymer, with a 32% HMF yield, 12% EMF yield, and 2% ethyl levulinate yield. The explanation behind the better performance of Purolite PD206, followed by Purolite CT275DR and finally both Amberlyst-15DRY and Purolite CT269DR, is the accessibility of the reactants to the active sites. Both Amberlyst-15DRY and Purolite CT269DR display the lowest pore volume among the macroreticular polymers. Purolite PD206, on the other hand, has been shown to swell well beyond the capacity of the other macroreticular polymers, generating very high porosity while in the reaction media. Thus, the differences detected among different catalysts are explained by the accessibility of the reagents to the sulphonic groups of the sulphonated polymers.

The results clearly indicate that the sulfonic groups supported over the polymeric matrix are much more effective in both the dehydration and etherification of fructose and HMF than protonic zeolites, improving the carbon balance of the reaction. This can be explained by the stronger acidity of sulphonic groups compared to zeolitic materials. Using the Gran potentiometric method, the acid sites of the zeolites were found to possess pK_a_ values ranging from 3.2 to 9.2 depending on the specific site type, chemical composition, and framework structure [18]. This contrasts with the strong acidity exhibited by the sulfonic acid groups in the investigated polymers, for which their acid sites possess pK_a_ values ranging from −2 down to −10 depending on the substituents of the aromatic ring [19]. Moreover, accessibility may be a limiting factor, as the microporosity of zeolites can hinder the diffusion of bulkier molecules such as fructose. On the other hand, the pores of the investigated sulfonated materials are on the order of micrometers; therefore, accessibility constraints in these polymers were absent. Moreover, it should be noted that resins swell with the solvent, increasing their surface area and accessibility towards their acid sites. Purolite PD206 has been reported to present a higher swelling ratio than other investigated sulphonated materials [20]. The gel-type structure of the Purolite PD206 resin exhibited significantly higher EMF and HMF yields than the other macroreticular polymers. This suggests that the swelling capacity of the gel-type Purolite PD206 offers a key advantage in terms of active site accessibility, which positively affects furanic yields during the reaction [20,21].

**Figure 2:**
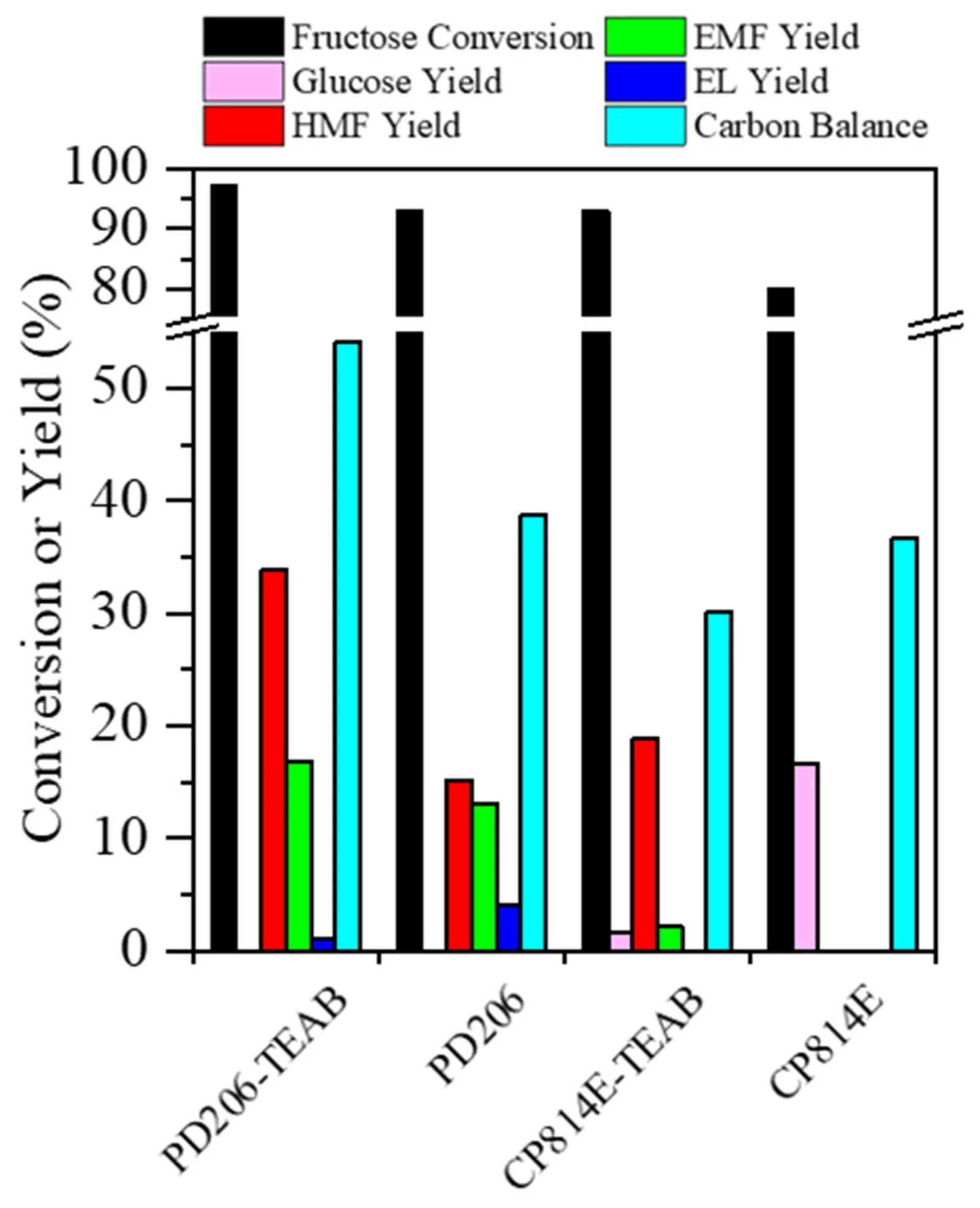
Effect of TEAB in the dehydration of fructose using Purolite PD206 and CT814E (10 mL of ethanol; 1.25 g fructose; when indicated, fructose:TEAB at a molar ratio of 6:1; 0.125 g catalyst; 100 °C; 5 h).

Thus, it has been demonstrated that sulphonated polymers are very effective catalysts in the transformation of fructose into furanics. To elucidate the effect of TEAB as a promoter of fructose dehydration and formation of furans, catalytic experiments were conducted in the absence of this salt using Purolite PD206 and the Beta zeolite CP814E (Figure 2).

These experiments revealed the strong promoter effect of TEAB in the production of EMF. The zeolite CP814E was unable to carry out the formation of HMF or EMF from fructose in the absence of TEAB, although the conversion of fructose was very high. This behavior is consistent with the formation of humins: the polymerization of the reagent and loss of carbon solubilized in the reaction medium. Also, without the HBA, glucose was the main product observed with a 17% yield. This observation aligns with the previous literature reporting the use of Br^−^ as a promoter for the isomerization of glucose into fructose. When TEAB is employed, Br^−^ facilitates the reconversion of glucose into fructose for its later dehydration with the help of zeolitic acid sites [22,23]. In contrast, the presence of TEAB significantly shifts the reaction’s selectivity, favoring HMF (19% yield) with only minor amounts of EMF produced. A similar trend was observed when using the gel-type sulfonated Purolite PD206; in the absence of TEAB, Purolite PD206 yielded 15% HMF and 13% EMF. Upon the addition of TEAB, the HMF yield more than doubled to 34%, while the EMF yield increased only slightly to 17%. This resulted in a substantial rise in total furanic yield from 28% to 51%. Notably, the fact that HMF production increased twofold while the EMF yield remained relatively stable suggests that TEAB primarily promotes the dehydration of fructose to HMF rather than the subsequent etherification step. Consequently, the HBA appears to facilitate fructose activation and its triple dehydration without participating in the conversion of HMF to EMF.

These results confirm that the addition of an HBA, such as TEAB, exerts a significant influence on the selectivity of fructose conversion in ethanol using acid catalysts. Regarding the role of the anion, the literature indicates that specific anions can promote fructose dehydration through several mechanisms, including hydrogen bonding and the stabilization of cationic intermediates [11]. Anions have been shown to exert a profound influence on the dehydration of fructose to HMF. For instance, Hu et al. leveraged micellar microreactors in dioxane to exploit the catalytic properties of bromide ions (Br^−^), which facilitate the dehydration process by assisting the deprotonation of key carbocationic intermediates [24]. Hu et al. attained up to a 70% HMF yield after just 15 min at 140 °C. Mankar et al. found an HMF yield of 14% when they employed a lactic acid-based DES. HMF yields increased to 87% after the incorporation of ChCl. Performing the same catalytic procedure with NaCl instead of ChCl allowed the obtention of a 70% yield of HMF, thus demonstrating that the presence of Cl^−^ was responsible for the high selectivity in fructose dehydration. Interestingly, in the literature, it has been reported that Cl^−^ has a positive effect on the selectivity towards HMF fructose dehydration, while Br^−^ salts show no significant change with respect to the absence of salt when performing the dehydration of fructose in 1-butanol while using HCl as a catalyst [25]. It has been reported that anions have a very limited effect in fructose dehydration when conducted in polar protic solvents [26]. This phenomenon is caused by anion solvation, which limits their interaction with fructose and its intermediates. On the other hand, when a polar aprotic solvent is employed, anions are less solvated, and their effect in fructose dehydration is dramatically higher. Moreover, Cl^−^ has been found to have more profound effects on the kinetics of fructose dehydration into HMF compared to Br^−^ and I^−^ [27]. At a mechanistic level, the Cl^−^ anion has a higher negative charge density with respect to Br^−^. As a result, Cl^−^ anions have stronger interactions with the positive charge density that is generated in the cationic intermediate species produced during fructose dehydration. Similarly, Cl^−^ anions are involved in the generation of hydrogen bonds, leading to their acting as stronger HBAs with more interactions with the HBD and fructose.

As the sulfonated Purolite PD206 behaved as the most promising catalyst in the transformation of fructose into high-value chemicals under mild reaction conditions, this study will delve into the optimization of the reaction conditions for Purolite PD206.

### 2.2. Optimization of fructose:HBA Molar Ratio

**Figure 3:**
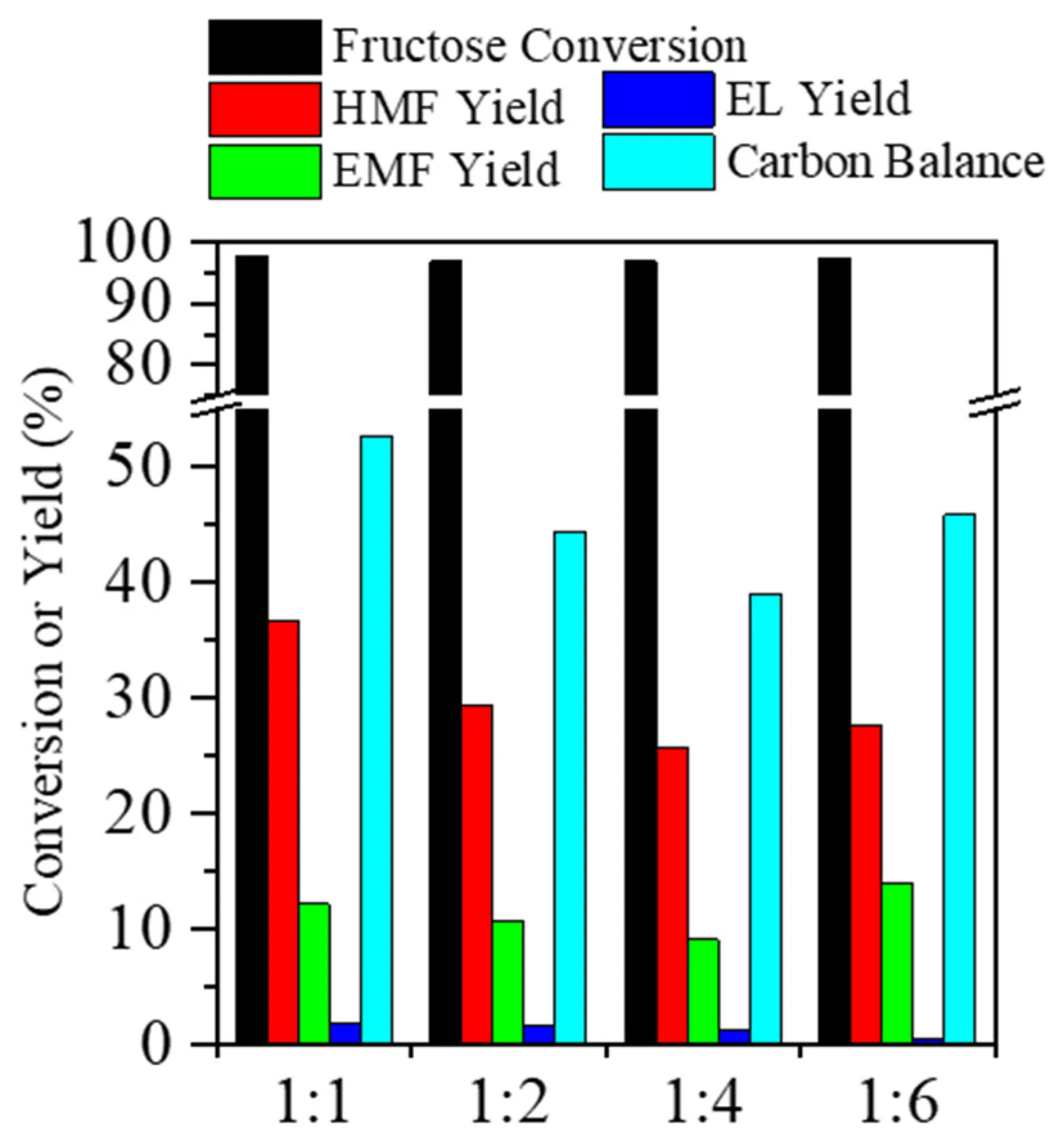
Effect of TEAB:fructose molar ratio in the dehydration of fructose (10 mL of ethanol; 1.25 g fructose; 0.125 g Purolite PD206; 100 °C; 5 h).

The effect of the fructose-to-HBA molar ratio was evaluated in the range of 6:1 to 1:1 (Figure 3). HMF yields changed from 27% when the 6:1 fructose:TEAB ratio was employed and up to 37% when a 1:1 fructose:TEAB ratio was utilized. Interestingly, EMF yields were mostly unaffected in all experiments. These results reinforce the idea that TEAB participates in the dehydration of fructose, stabilizing intermediates in the dehydration pathway of fructose.

The catalytic results demonstrate that the dehydration of fructose becomes more favorable when the TEAB:fructose ratio was close to 1:1. Specifically, HMF yields changed from 27% when the TEAB:fructose ratio was 1:6 to 29% and 37% when the ratio increased to 1:2 and 1:1, respectively. This trend is consistent with the participation of Br^−^ in the reaction system, interacting with fructose moieties to facilitate dehydration through the stabilization of fructose dehydration intermediates while EMF yields remain almost constant independently of HMF yields due to their lack of influence on etherification activity. Therefore, the 1:1 TEAB:fructose ratio was considered the most optimal for obtaining the highest furanic yield from the conversion of fructose in ethanol.

### 2.3. Time Optimization

**Figure 4:**
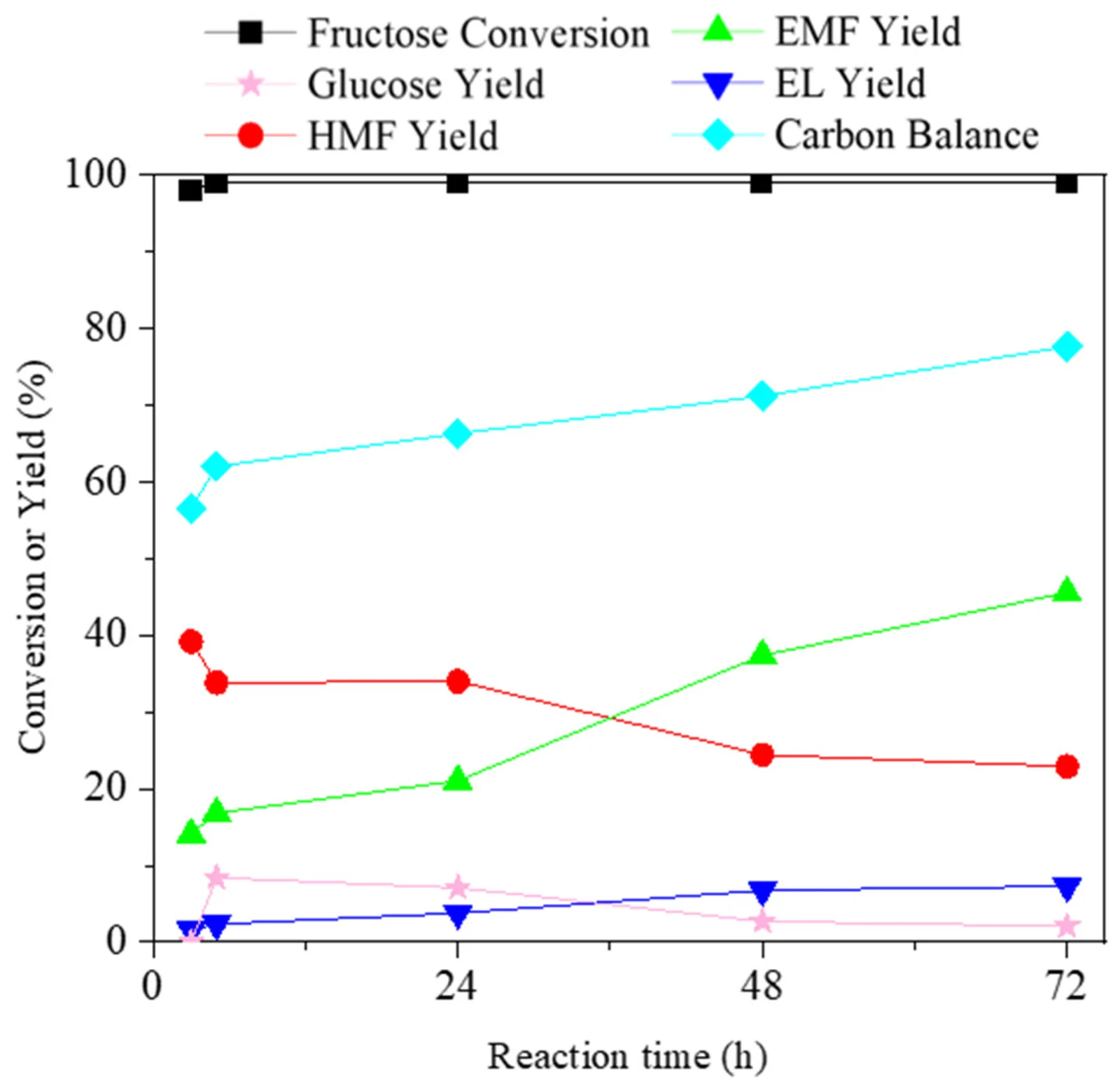
Conversion of fructose into different products with respect to reaction time (10 mL of ethanol; 1.25 g fructose; fructose:TEAB at a molar ratio of 1:1; 0.125 g Purolite PD206; 100 °C).

The evolution of fructose dehydration with time using Purolite PD206 has also been investigated (Figure 4). The activity of the catalyst in the transformation of fructose is highlighted by the 99% fructose conversion after just 3 h. As expected, a relationship can be identified in the observed yields of HMF and EMF. After just 3 h, HMF yields were as high as 39%, but they decreased as they were transformed into EMF. The HMF yield was 23% after 72 h of reaction. In contrast, the EMF yield increased from 14% at 3 h to 46% after 72 h. It can be deduced that fructose transformation takes place quickly, transforming fructose into furans, while the etherification of HMF is a slower process.

Moreover, ethyl levulinate yields rise from 2% to 7% over the course of the catalytic transformation. Ethyl levulinate is the result of the ethanolysis of the furanic cycle, and it is another valuable biofuel additive of special interest that improves the cold flow properties of biodiesel mixtures and has higher energy density than ethanol [28,29]. The carbon balance increased from 57% to 77% over time. This trend suggests that certain undetected intermediates or reversibly adsorbed species formed in the early stages are gradually converted into primarily furanic products or levulinate byproducts. An example of a potential intermediate is the one identified by Amarasekara et al., (4*R*,5*R*)-4-hydroxy-5-hydroxymethyl-4,5-dihydrofuran-2-carbaldehyde [30], or the difructose anhydride reported by Huang et al. [31].

Levulinic acid and ethyl levulinate are known products from fructose dehydration in water and acidic media, respectively. Previously, Aslanlı et al. reported the dehydration of fructose into alkyl levulinates using DESs under microwave irradiation [32]. Their findings indicated that methanol outperformed ethanol as a solvent; specifically, with a ChCl-pTSA, they attained yields of 59% for ethyl levulinate and 76% for methyl levulinate at 140 °C for 20 min.

After 72 h, an HMF yield of 23% still remained as a result of equilibria between HMF and EMF. The difficulty of the full transformation of HMF into EMF lies in the etherification and hydrolysis equilibria generated when fructose and possible degradation products are dehydrated, releasing water molecules into the medium. This effect was confirmed with a water content of 10% (9 mL of absolute ethanol and 1 mL of deionized water) used as a solvent in the transformation of fructose into furanics with TEAB and Purolite PD206 (Figure S2). The addition of 10% water slowed the conversion of fructose and increased the carbon balance (99% and 54%, respectively, without water compared to 97% and 60% with 10% water). The higher water content resulted in the more difficult etherification of HMF, which is translated into higher HMF yields (34% without water and 46% with water) and lower EMF yields (17% without water and 9% with water).

### 2.4. Effect of Pressure on the Product Distribution

**Figure 5:**
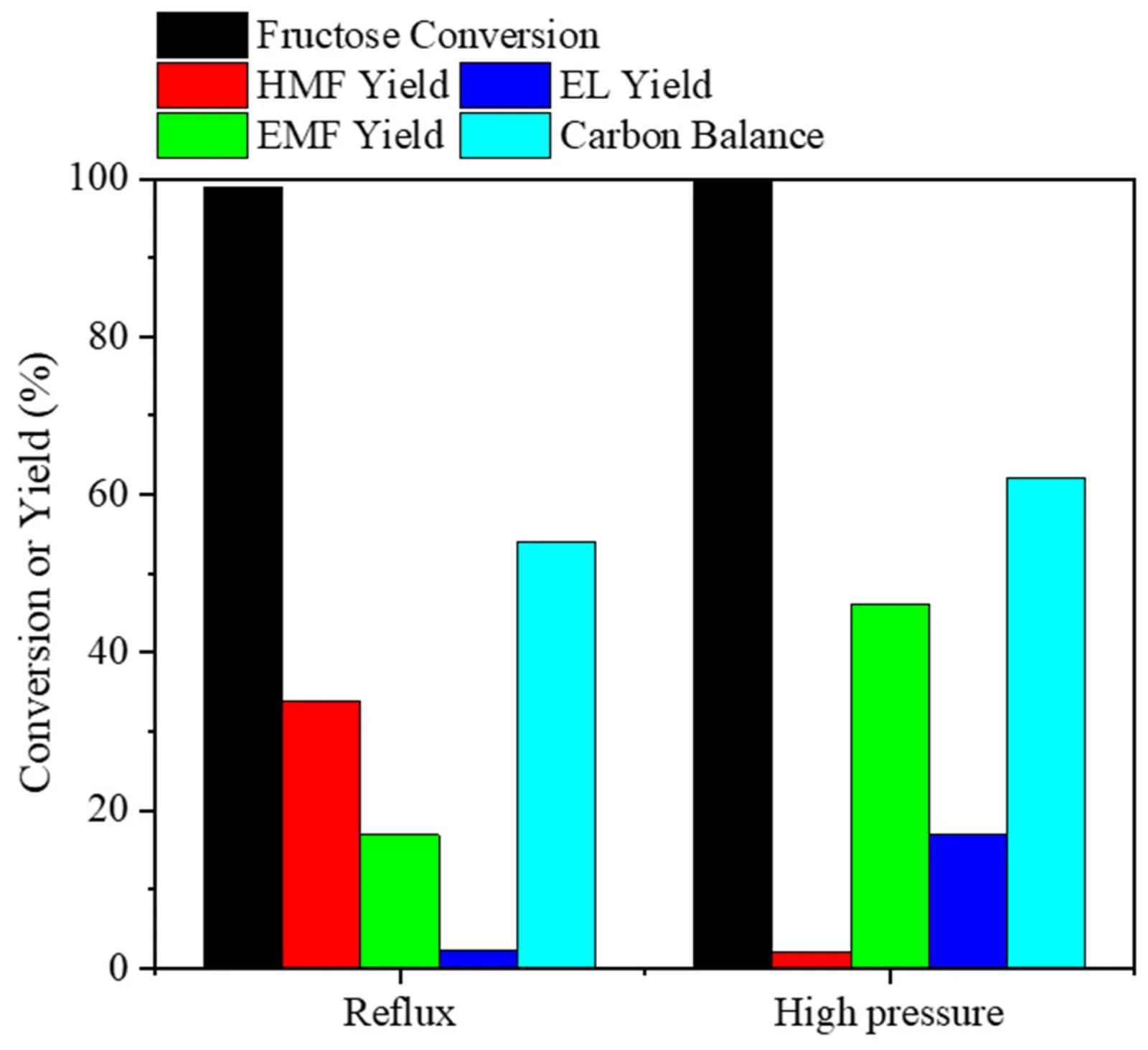
Comparison of the conversion of fructose and product distribution when utilizing a reactor under reflux (Quick-Thread Glass Reaction Tubes) and a pressurized reactor with autogenous pressure (Threaded Bush Ace Glass Pressure Tube) (10 mL of ethanol; 1.25 g fructose; fructose:TEAB at a molar ratio of 1:1; 0.125 g Purolite PD206; 100 °C; 5 h).

Previous experiments were performed at atmospheric pressure and under reflux. The behavior of the catalytic environment under autogenous pressure has also been investigated (Figure 5). While fructose conversion remained nearly quantitative in both setups, the distribution of furanic products changed dramatically. In the open, refluxing system, HMF was the primary product with a yield of 42%. Conversely, HMF was almost entirely consumed when working under autogenous conditions in a sealed reactor, with residual HMF yields dropping to a mere 2%. In parallel to the reduction in HMF yield, the yields of EMF and ethyl levulinate surged to 46% and 17%, respectively, under autogenous conditions. Furthermore, the increase in ethyl levulinate yields from 9% to 17% under autogenous pressure suggests that higher temperatures dramatically affect the HMF-EMF equilibrium, facilitating the ethanolytic ring opening of the furanic ring. Consequently, while operating at atmospheric pressure results in a diverse mixture of furanic products, the autogenous system selectively promotes the formation of EMF and ethyl levulinate. This behavior is expected, as autogenous pressure allows the reaction to exceed the atmospheric boiling point of ethanol (78 °C) and reach the target operating temperature (100 °C). This results in an effective difference of 22 °C, which provides the system with sufficient thermal energy to overcome the activation barriers associated with both HMF etherification and the ethanolysis of furanic rings into ethyl levulinate.

### 2.5. Screening of HBA

**Figure 6:**
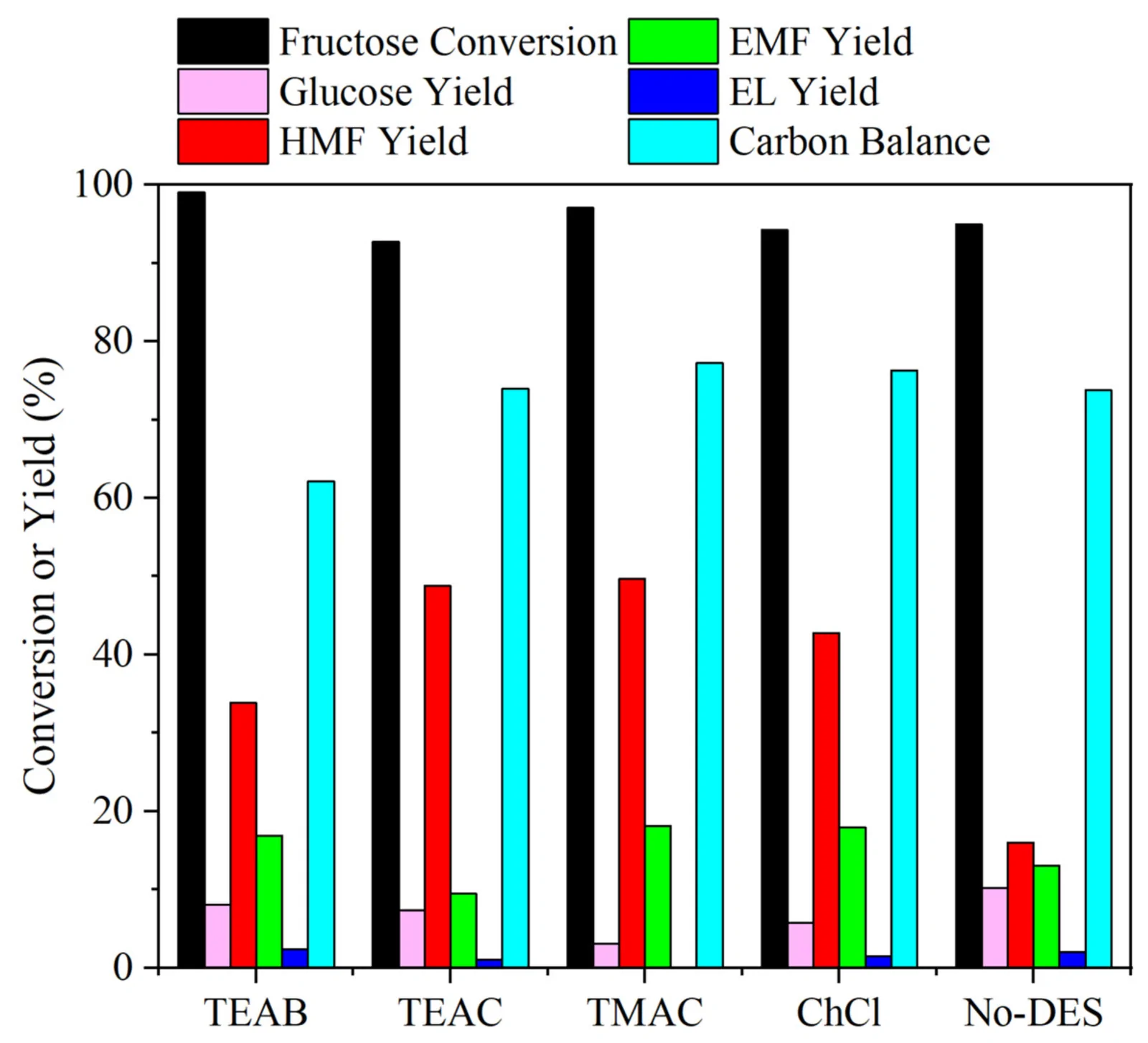
Conversion of fructose into different products employing different HBAs (TEAB, TEAC, TMAC, and ChCl) and no HBA (10 mL of ethanol; 1.25 g fructose; fructose:HBA at a molar ratio of 1:1; 0.125 g Purolite PD206; 100 °C; 5 h).

The positive effect of TEAB in the reaction media became clear after a substantial enhancement in HMF yield was observed (34% HMF yield when a fructose:TEAB ratio of 1:1 was employed) compared to the blank test without HBAs (15% HMF yield). Therefore, several other HBAs have been tested in the transformation of fructose into HMF and EMF, using a similar fructose:HBA ratio to assess the effectiveness of different cation and anion mixtures (Figure 6). In our approach, TEAB served as a model HBA to establish the optimal reaction parameters. Thus, we can conclude that distinct responses in the investigated solutions are caused by the different anions and cations utilized. 

Comparisons of the selected HBAs for fructose conversion in ethanol revealed a consistently positive effect on furanic yields, particularly for HMF. While the HBA-free control experiment yielded only 16% HMF, the addition of TEAB increased this to 34%. Superior results were achieved with chloride-based HBAs, where TEAC, TMAC, and ChCl reached HMF yields of 49%, 50%, and 43%, respectively. These findings suggest that, under the investigated conditions, Cl^−^ anions promote fructose dehydration more effectively than Br^−^ anions. In contrast to the significant boost in HMF production, the impact on EMF selectivity was more subtle; for instance, TEAC slightly reduced EMF yields compared to the HBA-free system. Nevertheless, total furanic yields (HMF + EMF) improved significantly from 29% (control) to 51% with TEAB and up to 58%, 68%, and 61% with respect to TEAC, TMAC, and ChCl, respectively, after 5 h at 100 °C. Finally, the presence of HBAs moderately suppressed the isomerization of fructose to glucose, reducing the glucose yield from 10% (only Purolite PD206, without HBA) to as low as 3% with TMAC.

The results agree with previous reports in the literature indicating that halides such as Br^−^ can promote glucose isomerization to fructose, facilitating the conversion of the saccharide moieties that are transformed into aldoses during the reaction system, which are more difficult to dehydrate [22]. In addition to this, the use of Cl^−^-based HBAs does not have a negative effect on the total carbon balance of the reaction with respect to the experiment in the absence of HBAs (74 to 77% Carbon Balance), while the use of TEAB resulted in a slight but notable decrease in carbon balance to 62% after 5 h of reaction.

In the literature, the influence of anions and cations has been discussed [26]. The use of Cl^−^ has been shown to have a small effect in fructose dehydration when water was utilized as solvent, but it has been demonstrated that it can increase the kinetics of fructose dehydration into HMF tenfold when using a polar aprotic solvent, much higher than the use of bromide [27]. This agrees with the behavior found in this work. Liang et al. investigated the application of different Al salts and found a correlation between the energy difference between the HOMO of an anion and the LUMO of glucose [33]. Br^−^, the anion with the lowest energy difference, resulted in the highest HMF yield, with an HMF yield of 74% (using AlBr_3_). Using anions with a higher energy difference, *Cl (74% HMF Yield), SO_4_^2−^ (61% HMF yield), and NO_3_^−^ (15% HMF Yield), resulted in progressively lower HMF yields. Thus, their group demonstrated that, under a biphasic system consisting of THF and a NaCl aqueous solution, Br^−^ provides significantly higher HMF yields. Wu et al. investigated different K salts to study the effect of different anions in the hydrothermal decomposition of glucose and fructose [34]. Their tests showed that Cl^−^ and Br^−^, together with I^−^ and NO_3_^−^, did not change the behavior of the reaction process significantly compared to the blank without the addition of salts when glucose was treated at 180 °C in water. Nevertheless, the mentioned anions did show a small improvement in the dehydration of fructose in a water environment. It is important to point out that even if fructose conversion was enhanced by the presence of Cl^−^, Br^−^, I^−^, and NO_3_^−^, the HMF selectivity found when anions were utilized was lower than blank tests without added salts. Thus, the benefits of employing anions in the production of HMF from glucose or fructose are heavily dependent on the reaction environment and conditions.

In this sense, other research groups also investigated the effects of ammonium salts in HMF production from saccharides. Tian et al. also investigated the effect of several ammonium halide salts for the dehydration of glucose into HMF [35]. They found that, generally, Br^−^ salts perform better in glucose dehydration than Cl^−^ while using SnCl_4_ salts as an acid catalyst. However, no clear trend could be observed when comparing the effects of different alkyl ammonium cations. Therefore, a complex set of interactions seems to govern the potential influence of the cations, requiring case-by-case investigation to confirm the most beneficial HBA for the dehydration process.

Ma et al. utilized TEAB in the direct production of 5-methoxymethylfurfural [36]. Among their findings, they report that Br^−^ and Et_4_N^+^ contribute to stabilizing charge separations during the formation of reaction intermediates from fructose, resulting in greatly improved yields. Using a mixed dimethyl carbonate–methanol solvent, a 77% yield of 5-methoxymethyl furfural was achieved. Cao et al. investigated the effects of different tetraalkylammonium salts in the dehydration of fructose into HMF [37]. They performed the dehydration of fructose at 120 °C for 70 min using a salt-to-fructose ratio of 2. The nine different tetraalkyl chloride salts originated from HMF yields between 60% and 81%, with TEA^+^ being the most effective salt among the investigated cations.

### 2.6. Use of TMAC Against Blank Test

**Figure 7:**
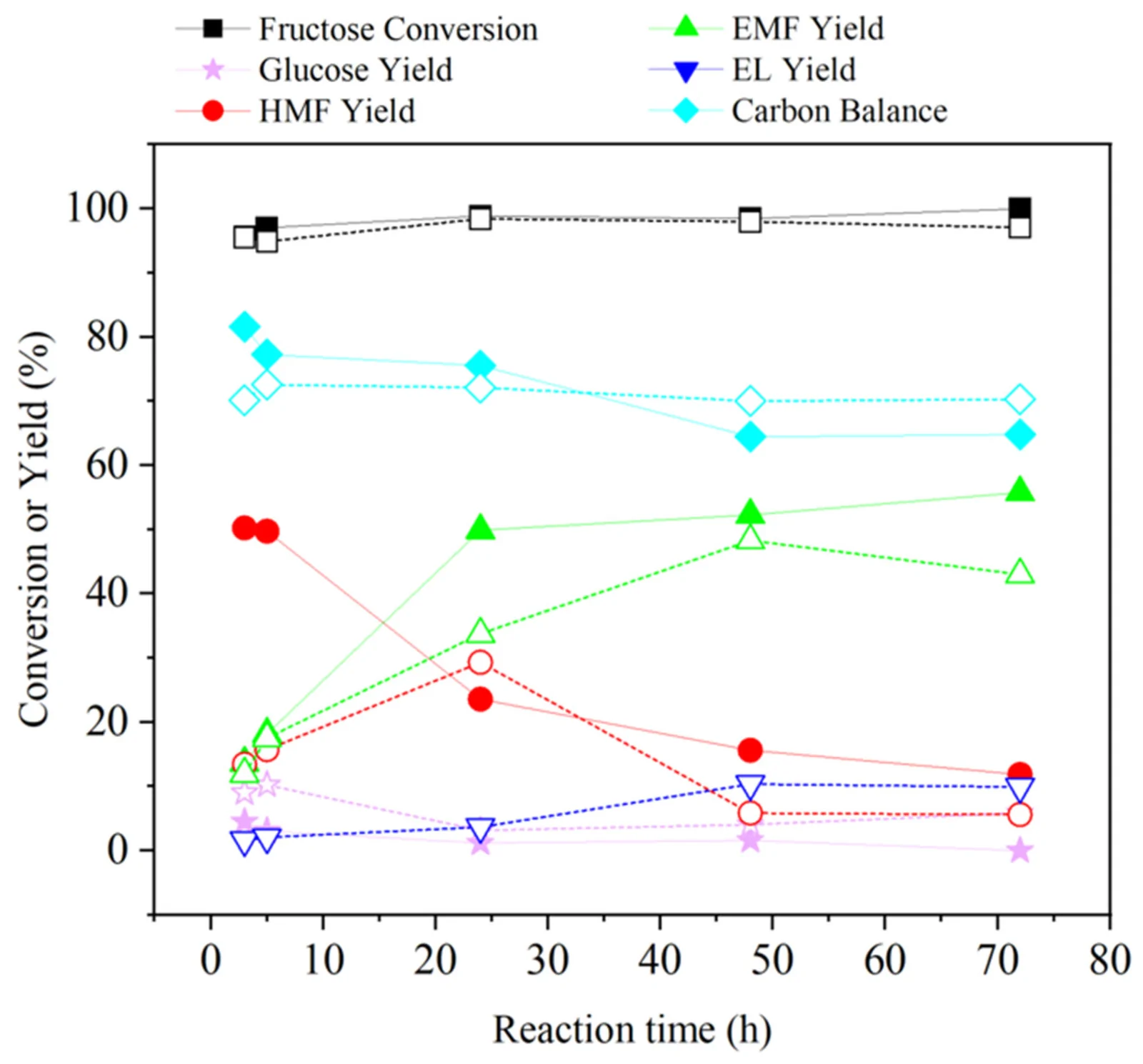
Conversion of fructose into different products employing TMAC as the HBA (continuous lines, full symbols) and no HBAs (dashed lines, empty symbols) with varying reaction times (10 mL of ethanol; 1.25 g fructose; fructose:TMAC at a molar ratio of 1:1; 0.125 g Purolite PD206; 100 °C).

Further research was conducted on the use of TMAC and its effect on the transformation of fructose after different reaction times compared with blank experiments without the addition of the HBA (Figure 7).

The investigation of fructose dehydration with respect to the reaction time shows the dramatic increase in the kinetics of the process with respect to the blank experiment, particularly at short reaction times. Below 5 h of reaction, a 50% HMF yield could be produced with TMAC, which was accompanied by 14% and 18% EMF yields after 3 and 5 h of reaction, respectively. This contrasts with the results observed after conducting fructose dehydration in the absence of HBAs, which yielded 13% and 16% of HMF after 3 and 5 h, respectively. Nevertheless, the EMF yield detected is very similar with or without the TMAC at lower reaction times. As the reaction progresses, the difference in selectivity towards EMF between both reaction systems widens. TMAC allowed the generation of an EMF yield up to 55% after 72 h at 100 °C. In the absence of HBAs, the EMF yield acquired was lower, with a maximum of 48% after 48 h (compared to 52% after 48 h of reaction). Moreover, HMF yield is also higher at longer reaction times when TMAC is utilized (12% HMF yield against 6% HMF yield). This suggests a strong stabilizing effect of the salt on the furanic ring of HMF while also strongly facilitating the dehydration of fructose.

The yield of glucose was 9% and 10% for the reaction system without HBAs after 3 and 5 h, respectively, after which it decreased below 5%. The yield of glucose was only 4% after 3 h when TMAC was used as the HBA, and it decreased to 0% after 72 h of reaction. The lower yield and the ultimate consumption of glucose when TMAC was used point to the influence of TMAC on the interconversion of glucose with fructose, facilitating the total consumption of all saccharides via the participation of sulphonic acids.

The promotion of fructose dehydration using an HBA was also investigated at 80 and 120 °C while under reflux (Table S2). The effect is clear, particularly at shorter reaction times, which shows HMF and EMF yields of 13% and 12% when not employing HBA, respectively, at 80 °C after just 3 h. On the other hand, the use of TMAC as HBA results in an increase in HMF and EMF yields up to 50% and 14%, respectively. Thus, the dehydration of fructose to HMF is increased dramatically at short reaction times. This translates to the faster transformation of HMF into EMF and higher total furanic yields at 24 h, with 24%, 50%, and 74%, respectively, when TMAC was employed and just 29%, 34%, and 63%, respectively, when no HBA was used. Furthermore, increasing the reaction time does not further improve the furanic yield of the experiments without HBA, as HMF and EMF undergo degradation. And very interestingly, the stability of EMF is enhanced when TMAC is present, as EMF yields continue to increase up to 56% after 72 h of reaction, in sharp contrast with the experiments lacking HBA, for which their EMF yields decrease from their maximum of 48% (after 48 h) to 43% (after 72 h). Increasing the reaction temperature to 120 °C while refluxing showed a similar scenario in which the dehydration of fructose in the absence of HBA is slow (16% and 21% of HMF and EMF yields, respectively, after 3 h) compared to experiments with TMAC (43% and 32% HMF and EMF yields, respectively, with a total furanic yield of 75% after 3 h). With the HBA, a maximum EMF yield of 53% could be attained after 24 h, after which it remains stable over time up to 72 h, while the EMF generated without HBAs undergoes degradation and decreases from 50% after 24 h to 40% at 72 h of reaction.

Therefore, the use of HBAs such as TMAC demonstrates a highly beneficial effect on the selectivity and activity of the transformation of fructose into valuable chemicals at low temperatures, which is beneficial from the point of view of the energy required to perform catalytic transformations. Eblagon et al. produced phosphorylated carbon catalysts that performed well in the dehydration of fructose to HMF. In this case, the stability of HMF was enhanced by the utilization of a non-oxidizing atmosphere, which was translated into an HMF yield as high as 73.3% in water after 3 h at 180 °C, aided by the participation of solubilized CO_2_ in the reaction media [38]. In our work, a furanic yield of 74% (HMF plus EMF) was attained after 24 h of reaction at 100 °C, and a furanic yield of 75% was attained after just 3 h at 120 °C. Thus, despite requiring longer reaction times, in this work, we report similar furanic yields at much lower temperatures, rendering the use of a high-pressure system unnecessary. Zhang et al. used sodium lignosulfonate waste, which was further upgraded through furfural condensation to increase the number of acid sites for the transformation of fructose into EMF in ethanol [39]. The protonated catalysts were very active in the production of EMF, achieving an EMF yield of 68.4% from fructose after its treatment for 40 min at 120 °C in ethanol. Regarding research works that also explored the optimization of anions and cations, Wrigstedt et al. tested CrCl_3_ as a catalyst for glucose dehydration [40]. Bromide improved the HMF yields obtained from glucose by enhancing fructose dehydration more effectively than Cl^−^ anions. They attained an HMF yield of 59% from fructose after 60 min, employing KBr, CrCl_3_, a mixture of water, acetone, and toluene at 140 °C. When glucose was used instead, a yield of 53% was reported. Their research found that both salts promoted a glucose-to-fructose ratio of 2:1 over time. Tong et al. [41] employed different salts to cocatalyze the dehydration of fructose into HMF in organic media. They investigated the activity of FeCl_3_ as an acid catalyst and different Cl^−^ and Br^−^ salts as promoters. Their findings suggest that Br^−^ salts promote an environment that is more beneficial for fructose dehydration than Cl^−^, which was ascribed to the generation of anionic iron complexes. Furthermore, the use of the large cation Et_4_N^+^ also positively impacted the HMF yield achieved during the reaction process, with FeCl_3_-TEAB attaining an HMF yield of 86% after 2 h at 90 °C, using N-methyl-2-pyrrolidone as solvent. Thus, our findings contrast with those reported by Tong et al., as not only Cl^−^-containing HBAs provided improved results with respect to TEAB, but the smaller TMA^+^ cation also had a positive effect on the product distribution of fructose transformation with respect to TEA^+^.

## 3. Materials and Methods

### 3.1. Materials

Absolute ethanol (99.96%) was acquired from VWR (Radnor, PA, USA). Tetraethylammonium bromide (TEAB) was purchased from Alfa Aesar (Heysham, UK) (98%). Fructose (99%), glucose (>99%), 5-hydroxymethyl furfural (HMF, >99.9%), 5-ethoxymethylfurfural (EMF, 97%), ethyl levulinate (99%), Amberlyst-15 (A15), tetramethylammonium chloride (TMAC, 97%), tetraethylammonium chloride (TEAC, 98%), and choline chloride (ChCl, 98%) were acquired from Sigma-Aldrich (St. Louis, MO, USA). Purolite PD206 (PD206), Purolite CT269DR (P269), and Purolite CT275DR (P275) were obtained from Purolite (King of Prussia, PA, USA). Zeolites CBV780, CBV5524, CBV2314, CP811C-300, CP814C, and CP814E were purchased from Zeolyst (Conshohocken, PA, USA).

Plotting has been performed through the manuscript employing Origin 2025 10.2.0.196.

### 3.2. Catalyst Properties

The catalysts employed in this work have been fully characterized in previous works [20,42].

All zeolitic materials were employed in their protonic forms after the calcination of all samples at 450 °C for 4 h (heating ramp of 2 °C·min^−1^). The employed zeolites were previously characterized in a published work [42]. Zeolite properties are summarized in Table S1. The characterization results suggest that the materials have very high surface areas, with Lewis and Brønsted acid sites. Beta zeolites (CP814E, CP814C, and CP814C-300) display a three-dimensional internal porous structure with 12-atom rings that allow the diffusion of large reactants [43]. The internal porous channels show a diameter that varies between 5.3 and 6.1 Å. Similarly to Beta zeolites, the material CBV780 (USY zeolite, structure type FAU) has a three-dimensional porous structure with very large channels characterized by T12 rings with a diameter of 7.35 Å [43]. In contrast, CBV2314 and CBV5524 are ZSM5 zeolites that present MFI structures [43]. The channel structure displayed by the MFI has a T10 ring structure. The porous system of the MFI has tubular pores with a diameter between 4.4 and 4.7 Å, significantly lower than that of FAU and Beta zeolites; thus, there was difficulty in diffusing large reactants through its internal structure.

The four polymeric catalysts have similar dry weight capacities, indicative of similar sulphonation levels (4.7–5.2 Eq·Kg^−1^). As indicated by the suppliers, Amberlyst-15DR, Purolite CT275DR, and Purolite CT269DR are predominantly macroreticular, as they display permanent porosity sustained in dry conditions thanks to their rigid structure (Table 1). Amberlyst-15DR and Purolite CT269DR are the materials with the highest surface area: from 35 to 53 m^2^·g^−1^. Purolite CT275DR displays lower surface areas (20–40 m^2^·g^−1^), but it has bigger pores that could enhance diffusion compared to Purolite CT269DR and Amberlyst-15DRY (400 to 700 Å average pore diameter in CT275DR versus 250 to 425 Å in Purolite CT269DR and Amberlyst-15DR). Purolite PD206, on the other hand, has no measurable surface area because it has a compact internal structure that only generates porosity through swelling with solvents. Nevertheless, swelling tests show that the gel-type material, Purolite PD206, has a much higher swelling ratio than the investigated macroreticular polymers [20]. Thus, using ethanol as a solvent, the very large swelling phenomenon is translated into more exposed active sites during reactions and higher activity. Moreover, analyses determined that the S content of the material was comparable or higher than that of the other sulphonic polymers, despite the analysis provided by the supplier.

### 3.3. Catalytic Tests

The catalytic dehydration of fructose and the subsequent etherification of HMF into EMF was conducted in 20 mL Quick-Thread Glass Reaction Tubes. The contents of the tubes were vigorously stirred with a stirring bar and heated with a Carousel 12 Plus™ Reaction Station (Radleys, Saffron Walden, UK). To ensure refluxing within the glass tube, the upper section of the Carousel was cooled down using a mixture of water and glycerol recirculated through a cooler. The, 10 mL of ethanol was added to the reaction tubes as a reactant and cosolvent. Reactions were performed with 1.25 g of fructose and a content of HBA that follows a 6:1, 4:1, 2:1, or 1:1 molar ratio with fructose.

The comparative experiment conducted under autogenous pressure was carried out using a Threaded Bush Ace Glass Pressure Tube (ACE LASS INC., Vineland, NJ, USA) with a volume of 15 mL. The pressure tube was submerged in a thermally controlled aluminum block. The pressure tubes were loaded with the same amount of ethanol, fructose, and HBA as the Quick-Thread Glass Reaction Tubes.

### 3.4. Analysis and Quantification

After a reaction, samples were prepared for analysis as follows: 0.4 mL of the sample was taken, diluted in 2 mL of water, and microfiltered before being analyzed via liquid chromatography. The analysis was carried out using a JASCO HPLC (Hachioji, Japan). The mobile phase consisted of pure microfiltered water pumped by a quaternary gradient pump (PU-2089). Products were separated in a Phenomenex REZEX Ca^2+^ Monosaccharide (Torrance, CA, USA) column (300 mm × 7.8 mm) using a 0.4 mL·min^−1^ elution flow and heated in a column oven (CO-2065) at 70 °C. Separated products were quantified using both a refractive index detector (RI-2031-PLUS) maintained at 40 °C with positive polarity and a multiwavelength detector (MD-2015) monitoring the wavelength at 278 nm. Conversion, selectivity, and yield were calculated as follows:

(1) $${Conversion}_{Y}=\left(\left({mol}_{initialY}-{mol}_{finalY}\right)\right)\cdot {{mol}_{initialY}}^{-1})\cdot 100$$

(2) $${Selectivity}_{X}=({mol}_{finalX}\cdot ({mol}_{initialT}-{mol}_{finalY}))\cdot 100$$

(3) $${Yield}_{X}=\left(Selectivity_{X}\cdot Conversion_{Y}\right)\cdot 100^{-1}$$

## 4. Conclusions

The dehydration of fructose into HMF and its subsequent etherification into EMF was studied by screening sulphonated polymers and protonic zeolites as catalysts. Sulphonated polymers provided better carbon balance values and much higher EMF and HMF yields than zeolites, which presented severe difficulties in the etherification of the small amounts of HMF produced, and only Beta zeolites showed small EMF yield values after reaction. It was demonstrated that the use of TEAB as HBA was key in the generation of high yields of furanics, as the removal of TEAB in tests with Purolite PD206 resulted in halving the HMF yield (34% versus 15% HMF yield) and reducing the carbon balance (54% with TEAB compared with 39% without it). No HMF or EMF yield could be found when CP814E was used without TEAB, compared to a 19% HMF yield when TEAB was present. Thus, it is demonstrated that the use of an HBA in ethanolic systems has a deeply beneficial effect on fructose dehydration, lowering energy barriers further than by the presence of acid catalysts alone.

The ratio of fructose was optimized, with the best results for HMF and EMF yields found when the fructose-to-HBA molar ratio was 1:1. Moreover, the use of hermetic reactors heated at 100 °C provided much higher yields of EMF (46%) and ethyl levulinate (17%). This result is interesting, as it provides high quantities of biofuel additives, and it is explained by the higher energy of the solvent after its temperature increases to 100 °C, in contrast to refluxing systems, which maintain 78 °C as the maximum temperature of the reaction system. Nevertheless, the non-refluxing system was maintained to optimize furanic yields.

The investigation of different HBAs, specifically TEAC, TMAC, and ChCl, produced furanic yields of 58%, 68%, and 61% (HMF plus EMF), respectively, versus a 25% yield of furanics in the blank test after 5 h at 100 °C. The result points to Cl^−^ as a strong promoter for the dehydration of fructose, with higher selectivity towards HMF. In all cases, the addition of an HBA had a strong, positive effect on the dehydration of fructose. TMAC led to better performance in the catalytic transformation, which prompted us to investigate the dehydration of fructose with TMAC over different reaction times. Up to 73% furanic yield was obtained from TMAC after 24 h of reaction at 100 °C, with the maximum HMF yield of 50% after just 3 h of reaction and an EMF yield of 55% after 72 h at 100 °C. After studying different temperatures, it was further demonstrated that the use of HBA stabilizes furanics and facilitates fructose dehydration into HMF and EMF, and a 75% yield of HMF plus EMF could be attained after just 3 h of reaction. Thus, the use of HBA is highly beneficial in the dehydration of fructose compared to the absence of HBA.

## Figures and Tables

**Table 1:** Characterization of sulfonated polymeric catalysts as provided by their suppliers.

	Structure Type	Dry Weight Capacity (Eq·Kg^−1^)	Surface Area (m^2^·g^−1^)	Pore Volume (mL·g^−1^)	Average Pore Diameter (Å)
Amberlyst-15DRY	Macro-reticular	4.7 ^a^	53 ^a^	0.4 ^a^	300 ^a^
Purolite CT275DR	Macro-reticular	5.2 ^b^	20–40 ^b^	0.4–0.6 ^b^	400–700 ^b^
Purolite CT269DR	Macro-reticular	5.2 ^b^	35–50 ^b^	0.3–0.5 ^b^	250–425 ^b^
Purolite PD206	Gel ^b^	4.9 ^b^	-	-	-

^a^ Provided by DuPont^TM^ (Wilmington, DE, USA). ^b^ Provided by Purolite^TM^ (King of Prussia, PA, USA).

## Data Availability

The original contributions presented in this study are included in the article/Supplementary Material. Further inquiries can be directed to the corresponding authors.

## Supplementary Materials

The following supporting information can be downloaded at https://www.mdpi.com/article/10.3390/ijms27177836/s1.
