# Supplementary material for: Hydrogen Bond Acceptors Dramatically Promote Fructose Dehydration and Stability of Furanics Like 5-Hydroxymethylfurfural
Source: Int J Mol Sci. 2026 Sep 1;27(17):7836. doi: 10.3390/ijms27177836 (PMC13565934; doi:10.3390/ijms27177836)
Supplement: Supplementary file 1 [file ijms-27-07836-s001.zip › ijms-4504870-supplementary.pdf]

Hydrogen Bond Acceptors Dramatically Promote Fructose Dehydration and Stability of Furanics Like 5-Hydroxymethylfurfural

Benjamín Torres-Olea 1,2,3,, Gabriela Rodríguez-Carballo 1,2, Isamar González-Díaz 1, Cristina García-Sancho 1,4,*, Ramon Moreno-Tost 1,4 and Pedro Jesús Maireles-Torres 1,4*

1 Departamento de Química Inorgánica, Cristalografía y Mineralogía, Facultad de Ciencias, Universidad de Málaga, Campus Universitario de Teatinos s/n, 29071 Málaga, Spain

2 Instituto de Materiales y Nanotecnología (IMANA), Facultad de Ciencias, Universidad de Málaga, Campus Universitario de Teatinos s/n, 29071 Málaga, Spain

3 Catalysis and Energy Materials Group, IMDEA Materials Institute, Calle Eric Kandel 2, Getafe, 28906 Madrid, Spain

4 Instituto Interuniversitario de Investigación en Biorrefinerías (I3B), Universidad de Málaga, Edificio de Investigación Ada Byron, Arquitecto Francisco Peñalosa, 18, Campanillas, 29010 Málaga, Spain

** Correspondence: benjamin.torres@imdeamaterials.org (B.T.-O.); cristinags@uma.es (C.G.-S.)*

Supplementary information

Figure S1: Blank reaction of transformation of fructose in ethanol in a refluxing Quick–Thread Glass Reaction Tubes. (10 mL of ethanol, 1.25 g fructose, 100 ºC, 5 h)

Figure S2: Investigation of the effect of the water content, comparing the use of absolute ethanol against the addition of 10 wt.% water. (10 mL of solvent, 1.25 g fructose, 0.89 g TEAB, 0.125 g PD206, 100ºC, 5 h)

Table S1. Information of the zeolitic materials according to the supplier and IZA zeolite database.

Table S2: Catalytic transformation of fructose at 80 and 120 ºC comparing the use of TMAC and the lack of HBA.

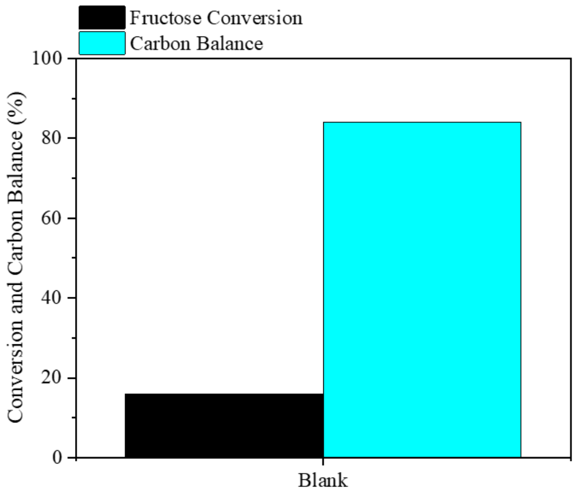

Figure S1. Blank reaction of transformation of fructose in ethanol in a refluxing Quick–Thread Glass Reaction Tubes. (10 mL of ethanol, 1.25 g fructose, 100 ºC, 5 h)

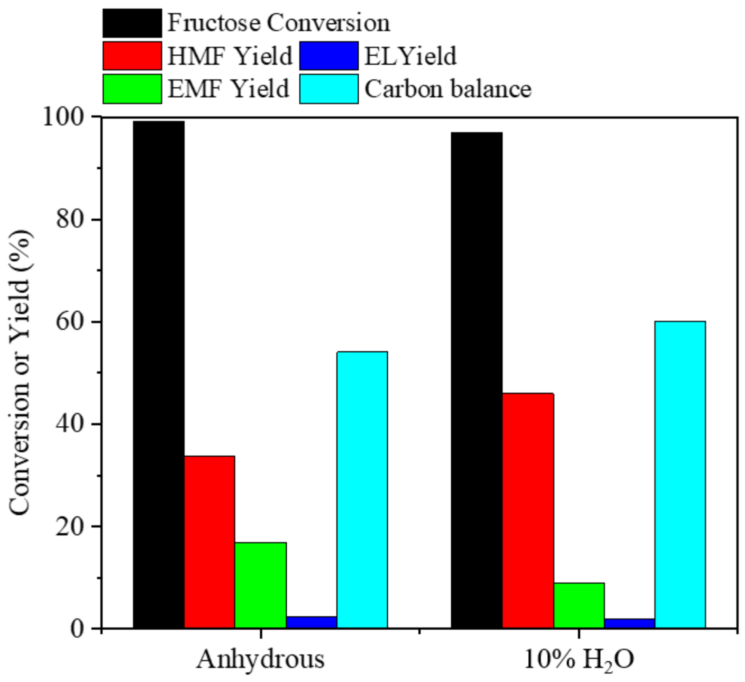

Figure S2. Investigation of the effect of the water content, comparing the use of absolute ethanol against the addition of 10 wt.% water. (10 mL of solvent, 1.25 g fructose, 0.89 g TEAB, 0.125 g PD206, 100ºC, 5 h)

Table S1. Information of the zeolitic materials according to the supplier and IZA zeolite database.

Zeolite name	Structure[a]	Cavity size (Å) [b]	Si to Al ratio[a]	Surface area ($m^2 \cdot g^{-1}$) [a]
CBV780	USY	11.24	40	780
CBV5524	ZSM5	6.36	5	425
CBV2314	ZSM5	6.36	11.5	425
CBV814-300	Beta	6.46-6.95	150	620
CP814C	Beta	6.46-6.95	19	710
CP814E	Beta	6.46-6.95	12.5	680

[a] Provided by Zeolyst™.

[b] Data from Database of Ordered Zeolite Structures, https://www.iza-structure.org/databases/.

Table S2. Catalytic transformation of fructose at 80 and 120 ºC comparing the use of TMAC and the lack of HBA.

Reaction type	Temperature (ºC)	Reaction time (h)	Fructose Conversion (%)	HMF Yield (%)	EMF Yield (%)
**No HBA**	80	3	96	13	12
**No HBA**	80	5	95	16	18
**No HBA**	80	24	98	29	34
**No HBA**	80	48	98	6	48
**No HBA**	80	72	97	6	43
**TMAC**	80	3	97	50	14
**TMAC**	80	5	96	50	18
**TMAC**	80	24	99	24	50
**TMAC**	80	48	99	16	52
**TMAC**	80	72	100	12	56
**No HBA**	120	3	96	16	21
**No HBA**	120	5	95	16	28
**No HBA**	120	24	98	9	50
**No HBA**	120	48	98	10	39
**No HBA**	120	72	97	6	40
**TMAC**	120	3	97	43	32
**TMAC**	120	5	96	36	37
**TMAC**	120	24	99	21	53
**TMAC**	120	48	99	17	45
**TMAC**	120	72	100	14	50
